# The Yabu Cohort Study: Design and Profile of Participants at Baseline

**DOI:** 10.2188/jea.JE20140065

**Published:** 2014-11-05

**Authors:** Hiroshi Murayama, Yu Nofuji, Eri Matsuo, Mariko Nishi, Yu Taniguchi, Yoshinori Fujiwara, Shoji Shinkai

**Affiliations:** 1 Research Team for Social Participation and Community Health, Tokyo Metropolitan Institute of Gerontology, Tokyo, Japan; 東京都健康長寿医療センター研究所 社会参加と地域保健研究チーム; 2 University of Michigan School of Public Health, Ann Arbor, MI, USA

**Keywords:** cohort profile, community-dwelling elderly, population approach, social relationship, Yabu Cohort Study

## Abstract

**Background:**

Further evidence into the effects of social relationships on health (including those at both the individual and community levels) is needed in Japan. The Yabu Cohort Study was launched in 2012 to identify the associations between social relationships and health among community-dwelling older Japanese people and to evaluate population approaches for preventive long-term care in the community. This report describes the study design and the profile of the participants at baseline.

**Methods:**

The Yabu Cohort Study is a prospective study of community-dwelling individuals aged 65 years and older in Yabu, Hyogo Prefecture, Japan. The baseline survey, using a mailed self-administered questionnaire, was conducted from July through August 2012. It included information on socioeconomic status, general and psychological health, and social relationships (social network, social support, and social capital). Survival time, long-term care insurance certification, and medical and long-term care costs after the baseline survey will be followed.

**Results:**

Of 7271 questionnaires distributed, a total of 6652 were returned (91.5% response rate), and 6241 were included in the analysis. Mean age was 71.9 ± 5.2 years, 43.2% were men, and 83.8% had lived in their neighborhood for more than 40 years. Approximately 45.2% expressed general trust. About 82.4%, 49.9%, and 55.5% have participated in neighborhood association activities, municipal seminars for preventive long-term care, and salon activities in the community, respectively.

**Conclusions:**

The study is expected to provide valuable evidence on the effects of social relationships on health and to suggest the usefulness of population approaches for preventive long-term care in Japanese communities.

## Abstract (ja)

**【背景】:**

個人レベルおよび地域レベルの社会関係が健康にもたらす効果について、日本では更なるエビデンスの構築が求められている。日本人地域在住高齢者の社会関係と健康の関連を検討し、地域におけるポピュレーションアプローチを評価するため、2012年に養父コホート研究を立ち上げた。本報では、養父コホート研究の研究デザインとベースラインにおける研究参加者の特性について記述する。

**【方法】:**

養父コホート研究は、兵庫県養父市に在住する65歳以上の高齢者を対象にした前向き研究である。2012年7月から8月の期間、郵送による自記式質問紙を用いたベースライン調査を実施した。質問紙には、社会的経済状態、全体的および心理的健康度、ソーシャルネットワーク、ソーシャルサポート、ソーシャルキャピタルをはじめとする社会関係、等の変数が含まれた。死亡、転出、介護保険認定、医療・介護給付費に関する情報は、ベースライン調査以降追跡されている。

**【結果】:**

7,271票配布された質問紙のうち、6,652票が回収され（回収率91.5%）、6,241票が分析対象となった。平均年齢は71.9±5.2歳、男性は全体の43.2%、現在住んでいる地区に40年以上居住する者は83.8%であった。45.2%が、一般的に人は信頼できると回答した（一方、37.7%はどちらとも言えない、17.1%は信頼できないと回答した）。町内会活動、市主催の介護予防セミナー、地区のサロン活動に参加したことがある者は、それぞれ82.4%、49.9%、55.5%であった。

**【結論】:**

養父コホート研究は、社会関係と健康についての有益なエビデンスを生み、日本における地域でのポピュレーションアプローチの有効性を提示することが期待できる。

## INTRODUCTION

The relevance of social determinants of health has been globally recognized as “solid fact.”^1^^,^^2^ The social determinants of health are the conditions in which people are born, grow, live, work, and age. Research suggests that efforts to improve community health should focus on the background risks of illness and unhealthy conditions. Social relationships (e.g., the social network and support that individuals possess, and the social cohesion and social capital of the community) are considered part of these social determinants of health. A previous study using a meta-analysis reported that one’s social network and social support had comparable influence to well-established risk factors such as smoking and drinking on risk of mortality.^3^ Further, a review of the literature showed that stronger social capital at the community level generally appeared to have a positive influence on individuals’ health status.^4^

It is widely known that Japan, one of the countries with the highest longevity in the world, has a relatively collectivist society characterized by intense group ties within the community.^5^^–^^7^ Therefore, social relationships may influence not only people’s perceptions and behaviors but also their health status. Moreover, rural areas tend to be more collectivist than urban areas in Japan.^8^ This suggests that the characteristics of traditional Japanese society may remain stronger in the former context. Epidemiological studies can promote understanding of the effects of social relationships on health, and several Japanese longitudinal studies examining the link between social relationships and health outcomes have been published thus far.^9^^–^^13^ However, no study in a traditional rural community exists. Investigation in highly collectivist communities could enable comparison with findings from previous studies and contribute to understanding the mechanisms by which social relationships, both at the individual and community level, affect health.

Many cohort studies fail to pay attention to evaluating a population approach for preventive long-term care. To evaluate this approach, a community-based and long-term evaluation design with appropriate outcomes (such as certification of long-term care, medical/long-term care cost) should be prepared. However, few population studies have been evaluated in this way.

Given these considerations, we launched the Yabu Cohort Study in 2012 to prospectively identify the associations between social relationships and health among community-dwelling older Japanese individuals and to evaluate population approaches for preventive long-term care in the community. Here, we describe the study design and the profile of the participants at the baseline of the study. For the present report, we referred to a previous article on the design and the profile of participants at baseline of another cohort study.^14^

## METHODS

### Study design, setting, and participants

In this prospective study, the source population comprised all community-dwelling people aged 65 years and older in the city of Yabu, Hyogo Prefecture, Japan. The baseline survey was conducted from July through August 2012 using a mailed self-administered questionnaire. Figure depicts a flow diagram of study participants. As of June 1, 2012, the population of people aged 65 years and older was 8673. After people with long-term care insurance (LTCI) certification levels 1–5 (i.e., people who require long-term care services; *n* = 1386) and those who died between June 1 and the date of the questionnaire dispatched in July (*n* = 16) were excluded, a total of 7271 questionnaires (the eligible population) were distributed. To combine the data from the questionnaire survey with the data from the follow-up survey (described below), we added a label with identification number and participant’s name, sex, and address to the questionnaire. However, our researchers were able to use only anonymous data. Before the survey, local residents were informed about the survey through the Yabu City bulletin. A reminder was sent to non-respondents in August 2012 to reduce response bias.

**Figure.**
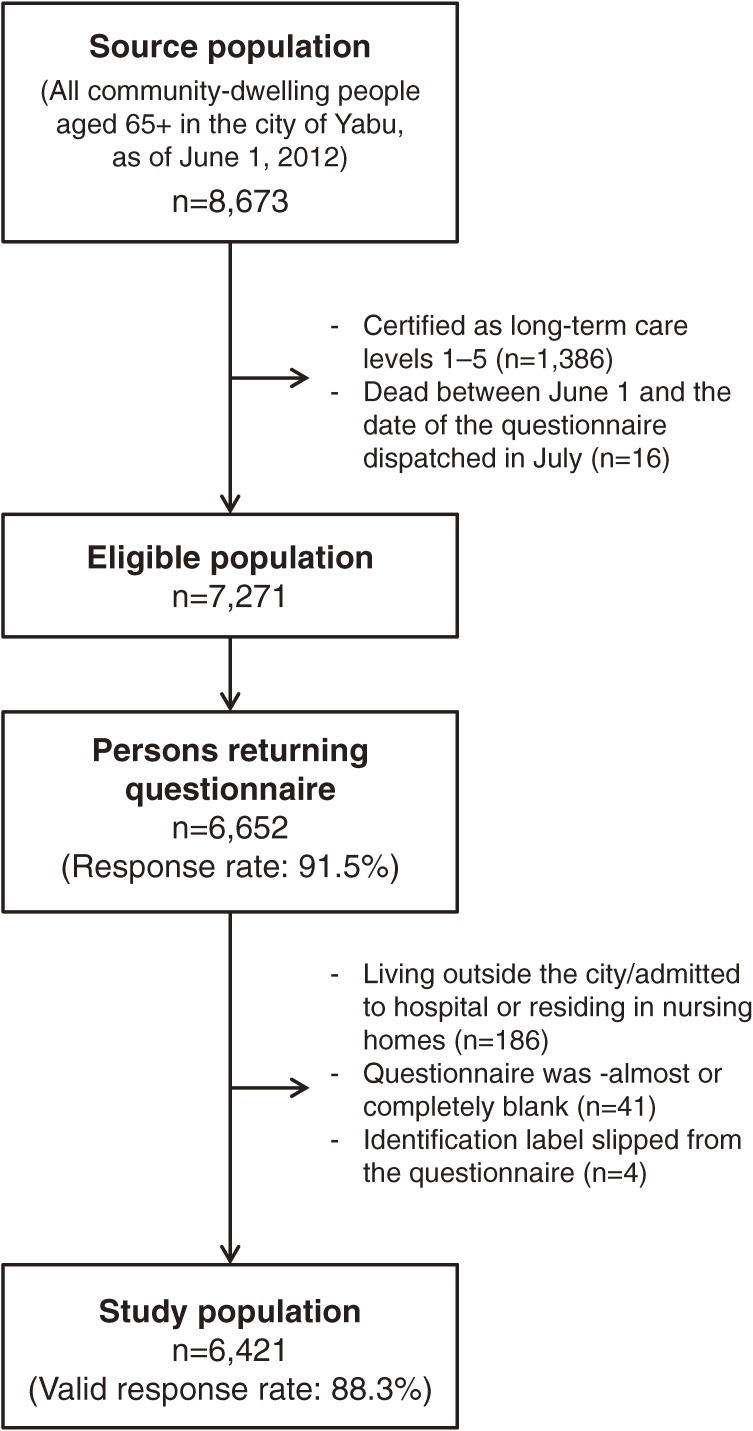
Flow diagram of study participants: the Yabu Cohort Study 2012

Yabu is located in the northern part of Hyogo Prefecture and is about 100 km northwest of Osaka and Kyoto. As of July 1, 2012, it had a population of 26 642 (12 787 were male and 13 855 were female) and a population density of 63.0 persons/km^2^. The proportion of people aged 65 years and older was approximately 32.6%. In Yabu, 8.2% of workers were employed in primary industries, 28.2% in secondary industries, and 63.0% in tertiary industries (compared to 4.2%, 25.2%, and 70.6%, respectively, at a national level).^15^ From April 2011 through March 2012, total numbers of inflow (people moving to Yabu) and outflow (people moving from Yabu) were 428 and 699, and the rates of inflow and outflow were 1.63% and 2.66% (compared to 4.21% and 4.20%, nationally), respectively.^16^

Yabu has focused on community-based population approaches for years. For example, Yabu has conducted preventive long-term care seminars for all community-dwelling elderly individuals to enhance the levels of knowledge and behaviors regarding preventive long-term care among its older population (such as the importance of exercise, proper nutritional intake, and social participation). Staff of the community comprehensive support center have traveled around all administrative neighborhoods to collaborate with local neighborhood associations on conducting the seminar smoothly and effectively. Further, the city has trained health volunteers and encouraged their activities in local neighborhoods. “Salon” activities have also been developed in many neighborhoods, and many volunteers work in their neighborhood salons to promote social interaction and social ties among the elderly.

### Baseline survey

Table 1 summarizes the main measures surveyed at baseline.

**Table 1.** Summary of items on the Yabu Cohort Study at baseline, 2012

**Demographic**
Living arrangement
Years of residence in the neighborhood area, birthplace
Socioeconomic status
**Medical and life-style profiles**
History of physician-diagnosed diseases
Smoking, drinking, regular exercise, etc.
Body mass index
Food intake frequency
**General and psychological health**
Self-rated health
Depressive mode (GDS short form)
Well-being (WHO-5 Well-being Index)
Subjective social isolation
**Activities of daily living**
Basic activities of daily living
Higher order competence of independence (TMIG-IC)
**Frailty**
Kaigo-yobo checklist
**Social relationships**
Social network, social support
Cognitive social capital (social trust, norm of reciprocity, etc.)
Structural social capital (social participation, etc.)
**Neighborhood environments**
Neighborhood social capital^a^
Neighborhood living environment (accessibility, safety, landscape, etc.)^a^

GDS, Geriatric Depression Scale; TMIG-IC, Tokyo Metropolitan Institute of Gerontology Index of Competence; WHO, World Health Organization.

^a^These items were created by aggregating the individual responses on social capital and living environments within the neighborhood.

We collected information on living arrangements, years of residence in the neighborhood, birthplace, and socioeconomic status. Socioeconomic status includes subjective poverty level, educational attainment, and annual household income. Other background information asked about in the questionnaire included history of physician-diagnosed diseases (hypertension, cardiovascular disease, cerebrovascular diseases, hyperlipidemia, and diabetes mellitus), smoking status, frequency of drinking, frequency of regular exercise, height, weight, and food intake frequency. Body mass index was calculated from height and weight (kg/m^2^). Food intake frequency included 10 kinds of foods (e.g., meats, vegetables, and fish).^17^

We also solicited information regarding self-rated health, depressive mood, well-being, and subjective social isolation. Self-rated health was assessed using the question “How would you rate your current overall health?” The 15-item Geriatric Depression Scale (GDS) short-form^18^^,^^19^ was used to assess depressive mood. A cutoff point of 5/6 was adopted, and a score of ≥6 indicated depressive mood.^19^ Well-being was assessed using the World Health Organization-Five Well-being Index (WHO-5).^20^^–^^22^ Subjective social isolation was assessed by the question, “How often do you feel that you are isolated from others?”

Activities of daily living were assessed, including basic ADL (BADL) and higher-order competence of independence. BADL was measured using five items: walking, eating, bathing, dressing, and toileting. To measure higher-order competence, we used the Tokyo Metropolitan Institute of Gerontology Index of Competence (TMIG-IC), which consists of three subscales: instrumental self-maintenance, intellectual activity, and social role.^23^

Frailty was assessed using the kaigo-yobo (preventive care) checklist to screen for frailty, which consists of 15 items.^24^^,^^25^ The index score range is 0–15, and a higher score indicates a greater likelihood of frailty. The cut-off point was set between 3 and 4, and a score of ≥4 was classified as frailty.

We assessed social relationships, including social network, social support, and cognitive and structural aspects of social capital. We asked about the frequency of contact with family/relatives and friends, apart from cohabitating family members, as a measure of social network. Social support included availability of instrumental support (“Is there anyone who can help you when you are in trouble?”), informational support (“Is there anyone who can give you information and knowledge that you need?”), emotional support (“Is there anyone who can share your worries and feelings?”), and appraisal support (“Is there anyone who can give you appreciation and encouragement?”). Cognitive social capital included social trust and norms of reciprocity. Structural social capital included social participation in several organizations (e.g., neighborhood association and hobby, sports, and learning groups). As all residents in Yabu belong to neighborhood associations, we asked whether they had ever participated in neighborhood association activities. In addition, we asked about their frequency of participation in municipal seminars for preventive long-term care and salon activities.

Neighborhood environments, including neighborhood social capital and neighborhood living environment, was assessed by asking about respondents’ perceptions of their local living environment using several items, including accessibility to non-residential facilities, safety, and landscape. Yabu consists of 161 administrative neighborhoods. We aggregated individual-level responses on social capital and living environment in every neighborhood as an indication of neighborhood-level environmental measures.

### Follow-up survey

We will follow four types of outcomes: survival time, LTCI certification, medical cost on national health insurance, and long-term care cost. To monitor these outcomes since the baseline survey, we will confirm information on the date of death or movement out of the study area, LTCI certification status (the date and the level of LTCI certification), and medical and long-term care costs every month. This information will be provided by the city of Yabu.

### Ethical consideration

The study protocol was reviewed and approved by the Ethical Committee of the Tokyo Metropolitan Institute of Gerontology (approved May 24, 2012). All participants gave informed consent prior to inclusion in the study: a statement attached to the questionnaire explained the purpose of the study, the voluntary nature of participation, and a promise of anonymity in the analysis. Returning the questionnaire was viewed as consent to participate in the survey.

## RESULTS

Of 7271 questionnaires distributed, 6652 were returned (91.5% response rate). Excluding 186 questionnaires from respondents who did not actually live in the city or were admitted to hospital or residing in nursing homes, 41 questionnaires that were almost or completely blank, and 4 with missing identification labels, 6421 were finally identified as the study population and included in the analysis at an individual level (88.3% valid response rate) (Figure).

Table 2 shows the baseline characteristics of participants. The mean age was 71.9 ± 5.2 years, and 43.2% of participants were men. About 83.8% had lived in their neighborhood area for 40 years or more, and 76.8% were born in the city. Regarding social relationships, 91.1% had contact with others apart from their family in a week, whereas the proportions of people receiving support varied by the types of support given. Women tended to receive more support than men. The proportions of people answering “yes” to general trust, trust in neighbors, and norms of reciprocity were 45.2%, 55.5%, and 51.0%, while those answering “no” were 17.1%, 12.5%, and 13.8%, respectively. About 82.4% had participated in the activities of their neighborhood association, and 38.3% belonged to hobby, sports, or learning groups. In addition, 49.9% had participated in municipal seminars for preventive long-term care and 55.5% in salon activities. These proportions were higher in women than in men.

**Table 2.** Baseline characteristics of participants by age and sex: the Yabu Cohort Study, 2012

			Total	Men aged 65–74	Men aged 75+	Women aged 65–74	Women aged 75+
**Number of individuals**			6421	1223	1550	1352	2296
**Demographics**							
	Age	(mean ± SD)	71.9 ± 5.2	69.1 ± 2.5	80.9 ± 4.7	69.1 ± 2.6	81.1 ± 5.0
	Sex	Men, %	43.2	—	—	—	—
	Number of family members	Living alone, %	13.5	6.5	8.4	12.4	21.5
		2, %	37.8	41.8	43.1	41.7	29.8
		≥3, %	48.7	51.7	48.5	45.9	48.7
	Marital status	Married, %	69.3	90.1	83.3	75.3	44.6
		Divorced/widowed, %	29.2	7.4	16.3	22.9	53.8
		Unmarried, %	1.5	2.5	0.4	1.8	1.6
	Years of residence in the neighborhood area	1–19, %	5.7	9.0	3.9	8.0	3.7
		20–39, %	10.5	16.8	6.3	16.1	6.5
		40–59, %	36.7	20.7	15.5	61.9	44.5
		≥60, %	47.1	53.5	74.3	14.0	45.3
	Birthplace	In current residential neighborhood, %	26.0	46.4	42.5	12.1	12.1
		In Yabu City but outside the neighborhood, %	50.8	38.1	47.3	49.9	60.6
		Outside Yabu City, %	23.2	15.5	10.2	38.0	27.3
	Subjective poverty level	Affluent, %	10.0	8.8	12.4	7.9	10.2
		Middle, %	62.0	55.1	63.1	61.7	65.3
		Poor, %	28.0	36.1	24.5	30.3	24.5
	Years of education	≤12, %	87.6	83.7	88.0	83.8	91.7
		≥13, %	10.5	14.8	10.2	13.6	6.5
		Unknown, %	1.9	1.5	1.8	2.5	1.8
**Medical and life-style profiles**							
	Smoking	Current, %	7.5	21.0	10.2	2.0	1.3
		Past, %	27.9	56.3	64.7	2.7	1.6
		Never, %	64.9	22.7	25.1	95.4	97.1
	Body mass index (kg/m^2^)	<18.5, %	9.2	5.8	9.6	6.7	12.6
		18.5–24.9, %	72.4	70.2	74.3	72.6	72.1
		≥25.0, %	18.4	24.0	16.1	20.8	15.3
**General and psychological health**							
	Self-rated health	Poor, %	37.4	32.7	43.6	28.7	41.1
	GDS (range: 0–15)	≥6, %	39.9	33.4	43.7	33.4	44.9
	Subjective social isolation	Yes, %	20.8	18.3	22.7	20.2	21.5
**Activities of daily living**							
	BADL	Independent^a^, %	92.0	97.5	91.1	96.8	86.5
	Instrumental self-maintenance (range: 0–5)	Full points, %	76.6	81.7	67.5	94.9	68.4
	Intellectual activity (range: 0–4)	Full points, %	67.4	69.5	66.6	78.8	59.5
	Social role (range: 0–4)	Full points, %	60.0	63.5	51.6	75.4	54.5
**Frailty**							
	Kaigo-yobo checklist (range: 0–15)	≥4 (frailty), %	27.5	16.6	34.6	14.6	38.9
**Social relationships**							
	Social network	Contact with others more than once a week, % (apart from family members living together)	91.1	89.9	88.2	94.3	91.7
	Social support	Receiving instrumental support, %	90.3	85.6	87.2	92.8	93.4
		Receiving informational support, %	86.8	83.1	82.2	91.2	89.4
		Receiving emotional support, %	83.9	77.3	76.5	90.5	88.5
		Receiving appraisal support, %	77.9	74.7	73.9	84.4	78.6
	General trust	Yes, %	45.2	48.0	53.0	38.4	42.5
		Neither, %	37.7	37.4	32.3	42.7	38.5
		No, %	17.1	14.6	14.7	18.9	19.0
	Trust in neighbors	Yes, %	55.5	51.6	62.3	45.5	59.6
		Neither, %	31.9	35.0	25.5	40.0	29.4
		No, %	12.5	13.4	12.2	14.4	11.0
	Norm of reciprocity	Yes, %	51.0	47.3	53.1	48.7	53.1
		Neither, %	35.2	39.1	32.6	39.2	32.2
		No, %	13.8	13.6	14.3	12.1	14.7
	Neighborhood association	Have participated in the activity, %	82.4	86.3	80.9	83.9	80.4
	Hobby, sports and learning groups	Belonging, %	38.3	28.3	36.4	50.8	37.4
	Municipal care prevention seminar	Have participated in the seminar, %	49.9	28.3	49.5	52.5	60.7
	Salon activity	Have participated in the activity, %	55.5	33.1	51.4	57.4	69.2

BADL, basic activities of daily living; GDS, Geriatrics Depression Scale; SD, standard deviation.

Missing values were removed.

^a^BADL independency indicated all five kinds of BADL activities were independent.

Table 3 shows the baseline neighborhood characteristics. After we excluded 9 neighborhoods that included 2 or fewer respondents, a total of 152 neighborhoods that had three or more respondents were included in the analysis at the neighborhood level. The mean number of respondents in each neighborhood was 47.6, with a range of 3–185. The mean population in the neighborhood was 170.1 ± 122.8 persons. Both neighborhood-level cognitive and structural social capital were widely distributed.

**Table 3.** Baseline neighborhood characteristics in relation to demographics and social capital: the Yabu Cohort Study, 2012 (*n* = 152)

			Mean ± SD	Min–Max	Proportion distributions				

					0.0– 19.9%	20.0– 39.9%	40.0– 59.9%	60.0– 79.9%	80.0– 100.0%
**Neighborhood demographics**									
	Population (persons)		171.1 ± 122.7	9–633	—	—	—	—	—
	Aging rate								
		% people aged 65 years and older	35.1 ± 10.6	1.7–83.3	9	101	39	2	1
**Neighborhood cognitive social capital**									
	General trust								
		% people who responded “yes” in the neighborhood	44.3 ± 12.5	0.0–100.0	4	44	91	12	1
	Norm of reciprocity								
		% people who responded “yes” in the neighborhood	50.6 ± 11.0	25.0–100.0	0	23	101	27	1
	Trust in neighbors								
		% people who responded “yes” in the neighborhood	54.8 ± 14.3	0.0–100.0	1	18	79	51	3
**Neighborhood structural social capital**									
	Neighborhood association								
		% people who responded “have participated in the activity” in the neighborhood	82.1 ± 15.3	0.0–100.0	3	0	4	39	106
	Hobby, sports and learning groups								
		% people who responded “belonging” in the neighborhood	36.4 ± 14.1	0.0–71.4	17	69	61	5	0
	Municipal care prevention seminar								
		% people who responded “have participated in the seminar” in the neighborhood	53.4 ± 19.1	0.0–100.0	6	30	55	51	10
	Salon activity								
		% people who responded “have participated in the activity” in the neighborhood	57.8 ± 20.4	0.0–100.0	4	24	48	53	23

SD, standard deviation.

## DISCUSSION

We have described the study design and baseline profile of participants in the Yabu Cohort Study, which was launched in 2012. We found relatively high levels of social capital in our study population compared with the populations in other Japanese cohort studies.^26^^–^^28^ Regarding cognitive social capital, approximately 45% and 51% of our participants answered that they have general trust and share norms of reciprocity, respectively. This can be contrasted to findings in another cohort study, which showed these proportions to be about 27% and 30%, respectively, in the general older population.^26^^,^^27^ In terms of structural social capital, about 82% had participated in the activities of neighborhood associations in our study, in contrast to reports that the proportion of participation in these activities was 30%–60% in previous generations.^28^ Because about 85% of our study participants have lived in their neighborhood for 40 years or more and about 77% were born within the city, they are likely to have strong social connections with other local people and organizations. However, although Yabu has intense community cohesion on average, the proportion of people with high social capital at the neighborhood level was widely distributed. This suggests that it is possible to compare the effect of neighborhood-level social capital on people’s health among the neighborhoods using multilevel analysis.

The Yabu Cohort Study has some strengths. First, our baseline survey was conducted for all community-dwelling elderly persons (except those with LTCI certification levels 1–5), and the response rate was high. Therefore, there should be relatively little possibility of selection or response bias. By linking the baseline and follow-up data, we were able to assess various associations and outcomes that will reflect the actual conditions of the source population, including an evaluation of the effect of using population-based approaches for preventive long-term care. Second, various measures were assessed in the baseline survey. We were therefore able to explore the effects of social relationships on health from several perspectives, allowing us to make recommendations about effective population-based health promotion and preventive long-term care strategies.

Several study limitations also warrant mention. First, we collected the data using a self-administered questionnaire. Particularly among the variables on social relationships, the items have not been adequately validated. Second, this study was conducted in one city in a rural area, so the target population was limited in size. Care should therefore be taken when generalizing the findings. However, our study focuses on a unique Japanese area with high community cohesion. Further, Yabu has a large proportion of elderly people. Therefore, our findings may indeed prove useful in developing health promotion and preventive long-term care strategies in other areas that have rapidly aging populations.

In conclusion, the Yabu Cohort Study was launched in 2012 to prospectively identify the association between social relationships and health among community-dwelling elderly Japanese and to evaluate population approaches for preventive long-term care in the community. Information on survival time, LTCI certification, and medical and long-term care costs will be collected in the planned follow-up survey.

## ONLINE ONLY MATERIAL

Abstract in Japanese.

## References

[1] World Health Organization. Closing the gap in a generation: health equity through action on the social determinants of health. Geneva: World Health Organization; 2008.

[2] Wilkinson R, Marmot M. Social deteminants of health: the solid facts. Copenhagen: World Health Organization Regional Office for Europe; 2003.

[3] Holt-Lunstad J, Smith TB, Layton JB. Social relationships and mortality risk: a meta-analytic review. PLoS Med. 2010;7:e1000316. 10.1371/journal.pmed.1000316 20668659 PMC2910600

[4] Murayama H, Fujiwara Y, Kawachi I. Social capital and health: a review of prospective multilevel studies. J Epidemiol. 2012;22:179–87. 10.2188/jea.JE20110128 22447212 PMC3798618

[5] Nakane C. Japanese society. Los Angeles: University of California Press; 1970.

[6] Yamagishi T, Cook KS, Watabe M. Uncertainty, trust, and commitment formation in the United States and Japan. Am J Sociol. 1998;104:165–94. 10.1086/210005

[7] Yamagishi T, Yamagishi M. Trust and commitment in the United States and Japan. Motiv Emot. 1994;18:129–66. 10.1007/BF02249397

[8] Yamazaki M. Japanese culture and individualism (Nihon bunka to kojin shugi). Tokyo: Chuokoron-sha; 1990.

[9] Aida J, Kondo K, Hirai H, Subramanian SV, Murata C, Kondo N, . Assessing the association between all-cause mortality and multiple aspects of individual social capital among the older Japanese. BMC Public Health. 2011;11:499. 10.1186/1471-2458-11-499 21702996 PMC3144463

[10] Aida J, Kondo K, Kawachi I, Subramanian SV, Ichida Y, Hirai H, . Does social capital affect the incidence of functional disability in older Japanese? A prospective population-based cohort study. J Epidemiol Community Health. 2013;67:42–7. 10.1136/jech-2011-200307 22760221

[11] Kondo N, Minai J, Imai H, Yamagata Z. Engagement in a cohesive group and higher-level functional capacity in older adults in Japan: a case of the Mujin. Soc Sci Med. 2007;64:2311–23. 10.1016/j.socscimed.2007.02.009 17412472

[12] Kondo N, Suzuki K, Minai J, Yamagata Z. Positive and negative effects of finance-based social capital on incident functional disability and mortality: an 8-year prospective study of elderly Japanese. J Epidemiol. 2012;22:543–50. 10.2188/jea.JE20120025 23117222 PMC3798567

[13] Murayama H, Nishi M, Matsuo E, Nofuji Y, Shimizu Y, Taniguchi Y, . Do bonding and bridging social capital affect self-rated health, depressive mood and cognitive decline in older Japanese? A prospective cohort study. Soc Sci Med. 2013;98:247–52. 10.1016/j.socscimed.2013.09.026 24331905

[14] Murayama H, Nishi M, Shimizu Y, Kim MJ, Yoshida H, Amano H, . The Hatoyama Cohort Study: design and profile of participants at baseline. J Epidemiol. 2012;22:551–8. 10.2188/jea.JE20120015 23117221 PMC3798568

[15] Ministry of Internal Affairs and Communications. The 2010 population census [cited 2014 March 1]. Available from: http://www.stat.go.jp/english/data/kokusei/index.htm

[16] Ministry of Internal Affairs and Communications. Report on Internal Migration in Japan [cited 2014 March 1]. Available from: http://www.stat.go.jp/english/data/idou/index.htm

[17] Kumagai S, Watanabe S, Shibata H, Amano H, Fujiwara Y, Shinkai S, . Effects of dietary variety on declines in highlevel functional capacity in elderly people living in a community. Nihon Koshu Eisei Zasshi. 2003;50:1117–24. 14750363

[18] Burke WJ, Roccaforte WH, Wengel SP. The short form of the Geriatric Depression Scale: a comparison with the 30-item form. J Geriatr Psychiatry Neurol. 1991;4:173–8. 10.1177/089198879100400310 1953971

[19] Schreiner AS, Hayakawa H, Morimoto T, Kakuma T. Screening for late life depression: cut-off scores for the Geriatric Depression Scale and the Cornell Scale for Depression in Dementia among Japanese subjects. Int J Geriatr Psychiatry. 2003;18:498–505. 10.1002/gps.880 12789670

[20] Awata S, Bech P, Koizumi Y, Seki T, Kuriyama S, Hozawa A, . Validity and utility of the Japanese version of the WHO-Five Well-Being Index in the context of detecting suicidal ideation in elderly community residents. Int Psychogeriatr. 2007;19:77–88. 10.1017/S1041610206004212 16970832

[21] Awata S, Bech P, Yoshida S, Hirai M, Suzuki S, Yamashita M, . Reliability and validity of the Japanese version of the World Health Organization-Five Well-Being Index in the context of detecting depression in diabetic patients. Psychiatry Clin Neurosci. 2007;61:112–9. 10.1111/j.1440-1819.2007.01619.x 17239048

[22] Bech P, Olsen LR, Kjoller M, Rasmussen NK. Measuring well-being rather than the absence of distress symptoms: a comparison of the SF-36 mental health subscale and the WHO-Five well-being scale. Int J Methods Psychiatr Res. 2003;12:85–91. 10.1002/mpr.145 12830302 PMC6878541

[23] Koyano W, Shibata H, Nakazato K, Haga H, Suyama Y. Measurement of competence: reliability and validity of the TMIG Index of Competence. Arch Gerontol Geriatr. 1991;13:103–16. 10.1016/0167-4943(91)90053-S 15374421

[24] Shinkai S, Watanabe N, Yoshida H, Fujiwara Y, Amano H, Lee S, . Research on screening for frailty: development of “the kaigo-yobo checklist”. Nihon Koshu Eisei Zasshi. 2010;57:345–54. 20666121

[25] Shinkai S, Watanabe N, Yoshida H, Fujiwara Y, Nishi M, Fukaya T, . Validity of the “kaigo-yobo check-list” as a frailty index. Nihon Koshu Eisei Zasshi. 2013;60:262–74. 23942023

[26] Hanibuchi T, Kondo K, Nakaya T, Shirai K, Hirai H, Kawachi I. Does walkable mean sociable? Neighborhood determinants of social capital among older adults in Japan. Health Place. 2012;18:229–39. 10.1016/j.healthplace.2011.09.015 22000345

[27] Ichida Y, Kondo K, Hirai H, Hanibuchi T, Yoshikawa G, Murata C. Social capital, income inequality and self-rated health in Chita peninsula, Japan: a multilevel analysis of older people in 25 communities. Soc Sci Med. 2009;69:489–99. 10.1016/j.socscimed.2009.05.006 19523728

[28] Kuriyama S, Nakaya N, Ohmori-Matsuda K, Shimazu T, Kikuchi N, Kakizaki M, . The Ohsaki Cohort 2006 Study: design of study and profile of participants at baseline. J Epidemiol. 2010;20:253–8. 10.2188/jea.JE20090093 20410670 PMC3900849

