# Supplementary material for: The Yabu Cohort Study: Design and Profile of Participants at Baseline
Source: J Epidemiol. 2014 Nov 5;24(6):519–25. doi: 10.2188/jea.JE20140065 (PMC4213228; doi:10.2188/jea.JE20140065)
Supplement: Abstract in Japanese. [file je-24-519-s001.pdf]

養父コホート研究：研究デザインとベースラインにおける研究参加者の特性

村山洋史、野藤悠、松尾恵理、西真理子、谷口優、藤原佳典、新開省二

東京都健康長寿医療センター研究所 社会参加と地域保健研究チーム

【背景】個人レベルおよび地域レベルの社会関係が健康にもたらす効果について、日本では更なるエビデンスの構築が求められている。日本人地域在住高齢者の社会関係と健康の関連を検討し、地域におけるポピュレーションアプローチを評価するため、2012 年に養父コホート研究を立ち上げた。本報では、養父コホート研究の研究デザインとベースラインにおける研究参加者の特性について記述する。
【方法】養父コホート研究は、兵庫県養父市に在住する 65 歳以上の高齢者を対象にした前向き研究である。2012 年 7 月から 8 月の期間、郵送による自記式質問紙を用いたベースライン調査を実施した。質問紙には、社会的経済状態、全体的および心理的健康度、ソーシャルネットワーク、ソーシャルサポート、ソーシャルキャピタルをはじめとする社会関係、等の変数が含まれた。死亡、転出、介護保険認定、医療・介護給付費に関する情報は、ベースライン調査以降追跡されている。
【結果】7,271 票配布された質問紙のうち、6,652 票が回収され（回収率 91.5%）、6,241 票が分析対象となった。平均年齢は 71.9±5.2 歳、男性は全体の 43.2%、現在住んでいる地区に 40 年以上居住する者は 83.8%であった。45.2%が、一般的に人は信頼できると回答した（一方、37.7%はどちらとも言えない、17.1%は信頼できないと回答した）。町内会活動、市主催の介護予防セミナー、地区のサロン活動に参加したことがある者は、それぞれ 82.4%、49.9%、55.5%であった。
【結論】養父コホート研究は、社会関係と健康についての有益なエビデンスを生み、日本における地域でのポピュレーションアプローチの有効性を提示することが期待できる。

キーワード：コホート研究、地域在住高齢者、ポピュレーションアプローチ、社会関係、養父コホート研究
